# Multifractal characterization of the Coniacian–Santonian OAE3 in lacustrine and marine deposits based on spectral gamma ray logs

**DOI:** 10.1038/s41598-020-71327-w

**Published:** 2020-09-01

**Authors:** Kouamelan Serge Kouamelan, Changchun Zou, Chengshan Wang, Konan Roger Assie, Cheng Peng, Ohouo Rebecca Mondah, Koffi Alexis N’dri, Eric Thompson Brantson

**Affiliations:** 1 Key Laboratory of Geo-Detection (China University of Geosciences, Beijing), Ministry of Education, Beijing, 100083 China; 2 School of Geophysics and Information Technology, China University of Geosciences, Beijing, 100083 China; 3 Institute of Earth Sciences, China University of Geosciences, Beijing, 100083 China; 4 School of Earth Science and Resources, China University of Geosciences, Beijing, 100083 China; 5 Petroleum Engineering Department, Faculty of Mineral Resources Technology, University of Mines and Technology, Tarkwa, Ghana

**Keywords:** Climate sciences, Ocean sciences, Solid Earth sciences

## Abstract

Limited to the Atlantic and its surrounding basins, the expression of the Coniacian–Santonian oceanic anoxic event (OAE3) was discovered in the non-marine Cretaceous Songliao Basin, Eastern Asia not long ago. In this study, based on spectral gamma ray logs data recorded in three basins, the self-similarity of the OAE3 was studied through the analysis of the scaling properties of thorium–potassium and thorium–uranium distributions both in marine and terrestrial environments using the multifractal detrending fluctuation analysis. The results indicate that, in both marine and terrestrial systems, the OAE3 intervals are characterized by their multifractal nature due to long-range correlation. However, the multifractal features of the studied OAE3 intervals are different in the three basins, although some common trends were observed. By comparing the degree of multifractality of the OAE3 deposits with the clay minerals and the redox conditions, it appears that the changes of the multifractal features are controlled by local changes such as clay mineralogy and redox conditions in both milieus under different sedimentation patterns. At all sites, the left side shortened spectrum of the thorium–potassium distribution suggests the presence of local fluctuations with minor amplitudes during the OAE3. Furthermore, the shortened singularity spectrum of the thorium–uranium distribution reflects the existence of small-scale fluctuations with large amplitudes at marine sites while in the non-marine Songliao Basin, the thorium–uranium distribution suggests the presence of local fluctuations with small amplitudes during the OAE3. Therefore, a more local behavior of the event is considered although the regional character is not neglected.

## Introduction

**Figure 1:**
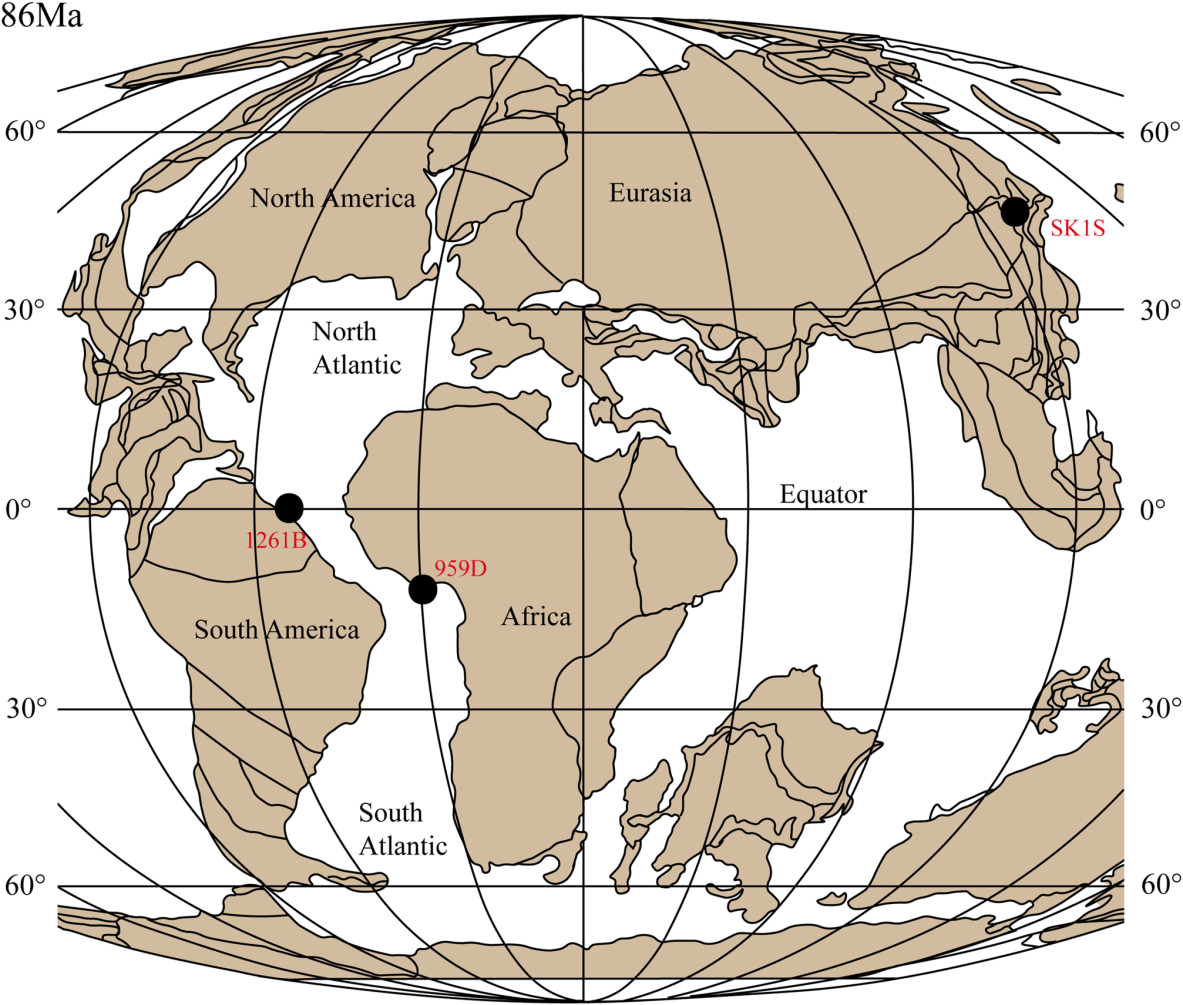
Site locations for the studied Coniacian–Santonian OAE in northeastern Asia, northwestern Africa and off south America (plate tectonic map of the Late Cretaceous for 86 Ma modified from Wagreich^56^). Location of sites 959D (Deep Ivorian Basin) and 1261B (Demerara Rise) are from Wagreich^56^; location of SK1S (Songliao Basin) is from Wang et al.^26^.

The Cretaceous is the last and longest period of the Mesozoic Era where the Earth experienced some major geological events such as oceanic anoxic events (OAEs)^1–5^, large-scale volcanic activities^5–8^ whose footprints are concealed in both marine and continental environments. OAEs have been documented in the geological sedimentary record, particularly in Cretaceous marine sediments, as complex paleoenvironmental phenomena and climatically influenced major geological perturbations of the Earth system, especially the Earth’s carbon cycle^4,9,10^. These complex geological events are characterized by global deposits of organic shales associated with major carbon isotope excursions (CIEs)^4,11–13^. Intensive investigations revealed that the Upper Cretaceous period recorded two important OAEs: (**1**) OAE2 (Cenomanian–Turonian) known as one of the severest and widespread oceanic anoxic events, and (**2**) OAE3 (late Coniacian–early Santonian) which represents the youngest Cretaceous oceanic anoxic event and potentially a regional rather than a global phenomenon, but which lasted much longer than OAE2^14–16^. Dissimilar to OAE2, the geographical distribution of the OAE3 suggests that the Coniacian–Santonian oceanic anoxic events are restricted to the Atlantic and proximate basins^10^ (Fig. 1). Although this event is characterized by significant burial of organic-rich shale, its exact paleo-ecological extent, spatial distribution and development are not well established^3,17–20^. However, most of these previous studies are one-sided i.e., firstly, they are focused on marine environments although the influence of OAEs in lacustrine environments has been described^4^. Secondly, a complete terrestrial-marine correlation of the last Upper Cretaceous OAE is not documented in the literature, mostly due to the fact that the terrestrial expression of the OAE3 (TEOAE3) is scarce. For instance, focusing on the equatorial Atlantic, Ocean Drilling Program (ODP) sites alongside the Deep Ivorian Basin (DIB) and the Demerara Rise (DR) precisely ODP Leg 159^21,22^ and ODP Leg 207^19,20^, which record this event. Although comparatively rare in lacustrine environments, the recent finding of the TEOAE3 in the upper Qingshankou in the non-marine Songliao Basin, northeastern China, suggests that paleo-lakes are proven suitable for testing hypotheses about the OAE3 triggering mechanisms in terrestrial environment^23^, thus opening a door for a possible terrestrial-marine OAE3 correlation. This is furthermore motivated by the work of Chamberlain et al.^24^ who found a synchronous response of OAE2 in the lower Qingshankou Formation in the Songliao Basin. So far, the Songliao Basin represents one of the nearly complete Cretaceous terrestrial records in the world^25^, making this area an optimal site to infer global, local or zonal paleoenvironmental changes.

Therefore, the question of whether there are similarities in the development of OAE3 in marine and terrestrial environments arises. Thus, carrying out a joint study of the Coniacian–Santonian oceanic anoxic event in both marine and lacustrine environments in different basins can help present a better understanding of the controlling mechanisms and its regional/local environmental responses in both marine and terrestrial systems. Moreover, previous studies mostly focused on organic proxies (biomarkers) and inorganic compounds including stable isotope compositions^26,27^. However, studies of the self-similarity of OAE3 based on petrophysical data have not been conducted.

In the geosciences, fractal and multifractal features have been increasingly used to describe geological events, geological processes and geological objects^28,29^. The principal advantage of fractal/multifractal theory concerns its capacity to characterize irregular and complex phenomena or processes that display similarity over a broad sequence of scales (self-similarity)^30^ that the traditional Euclidean geometry method (integral dimension) fails to analyze^31^. The fractal concept originates from geometric self-similarity which was first proposed by Mandelbrot^30^. Self-similar fractals evaluate the presence of an arrangement that is similar to itself on any scale. Since, the concept has been widely applied in medicine, geology, geophysics^28,31,32^. Many occurring natural processes like OAEs are complex phenomena with their development controlled by complicated processes, and thus do not follow normal distributions. Consequently, ordinary linear dataset analysis techniques are not sufficient for a comprehensive analysis of these events. The fractal method is one of the useful concepts to be applied to analyze complex systems^33–36^. Among numerous fractal methodologies developed since Mandelbrot’s findings, the concept based on the detrended fluctuation analysis (DFA) which was devised by Peng^37^ is the most frequently utilized. By expanding the concept of DFA, Kantelhardt et al.^38^ initiated the notion of multifractal detrended fluctuation analysis (MFDFA), which works similarly to the DFA. MFDFA is useful to reveal the multifractal behavior in any non-stationary data series. This method also allows to detect the causes of multifractality by comparing the estimated shuffled and surrogate data series derived from the original data series to the original data series^39–42^. Multifractality helps to describe more carefully and comprehensively the dynamic properties of systems, and characterize their behaviors both locally and globally^43^. MFDFA has been used by some scholars to study geophysical well log data^44,45^, which are governed by complex spatio-temporal dynamics of which non-linearity and scaling are the dominant processes^46^.

Using core samples from boreholes, previous studies have greatly improved our understanding of major Cretaceous marine and terrestrial changes^2,4–6,47^. However, integrated studies of marine and terrestrial Cretaceous deposits are rare^26^, and the multifractal correlation based on spectral gamma ray by means of well log data have not been conducted.

Compared to the core samples analysis, well logging has several advantages^48,49^. First, for executing high-precision continuous sampling (spectral gamma ray, nuclear magnetic resonance, etc.), and measurement of in-situ formation conditions. Second, for investigating a volume of sediment that is often greater than the one represented by a core or plug, and consequently than a cutting and so more representative of the mean properties of the rock, especially in heterogeneous rocks. Third, the human factors have little effects on the measurement processes. Among the well log data, the spectral gamma ray has proved particularly useful for understanding paleoenvironmental changes^50–53^.

To understand the multifractal behavior of the Coniacian–Santonian OAE, marine sedimentary records must be integrated with terrestrial deposits. Based on data from scientific drilling projects, this work can be achieved by correlating the non-marine Cretaceous Songliao Basin in eastern Asia with the marine Deep Ivorian Basin in western Africa and Demerara Rise off the coast of South America where a well preserved response of the OAE3 has been described^10,17,20,54–57^.

This paper is the first work integrating the multifractal nature of a marine and terrestrial OAE. The aim is to highlight and compare the multifractal properties of a rare and complex geological event such as OAE3 recorded in marine and terrestrial systems as well as analyze the causes of the multifractality and the possible factors affecting the multifractal features in both environments, which can help to understand the regional or local nature of the last Late Cretaceous OAE.

## Geological Setting of the drilling sites

### Songliao Basin (SLB)

**Figure 2:**
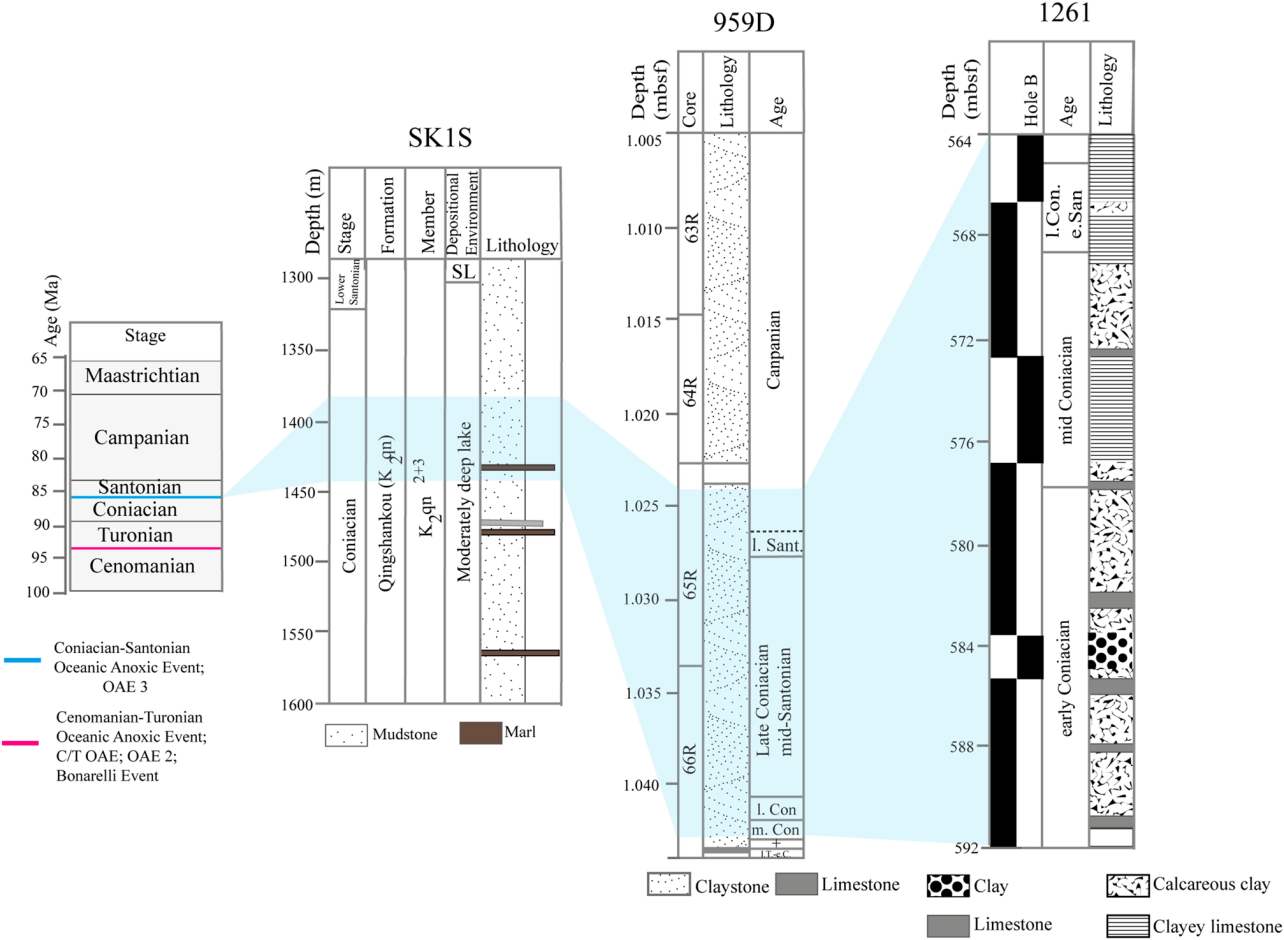
Geology of the studied Coniacian–Santonian OAE interval. The age and the location of the two Late Cretaceous OAEs are from Jenkyns^4^; the stratigraphy at sites SK1S, 959D and 1261B are modified from Wang et al.^63^, Beckmann et al.^57^ and Flögel et al.^89^, respectively.

The SLB is an elongated large non-marine Cretaceous basin located in northeastern China (Fig. 1). It represents a Mesozoic–Cenozoic intracratonic basin with the thickness of Cretaceous strata reaching up to 6,000 m^58,59^, making the SLB one of the largest and longest-lasting Cretaceous non-marine basins on the Earth^25^. A fluvio-deltaic and lacustrine sedimentary succession ranging from the Turonian to Campanian has been recorded through two scientific drilling sites (SK1S and SK1N). The Late Cretaceous formations at site SK1S consists of the older to the younger strata, of six formations namely the Quantou (${\text{K}}_{2} {\text{q}}$), Qingshankou (${\text{K}}_{2} {\text{qn}}$), Yaojia (${\text{K}}_{2} {\text{y}}$), Nenjiang (${\text{K}}_{2} {\text{n}}$), Sifangtai (${\text{K}}_{2} {\text{s}}$), and Mingshui (${\text{K}}_{2} {\text{m}}$) formations controlled by local tectonic motions and climatic variations^60,61^. ${\text{K}}_{2} {\text{qn}}$ is subdivided into lower ${\text{K}}_{2} {\text{qn}}$ (Member 1) composed mainly of organic-rich laminated mudstone and upper ${\text{K}}_{2} {\text{qn}}$ (Members 2 and 3) essentially formed of undifferentiated grey shales^62,63^, with presences of thin layers of colored marl in the lower section^58,59^. Members 2 and 3 of ${\text{K}}_{2} {\text{qn}}$ in hole SK1S are semi-deep lacustrine sediments, and mainly late Turonian to late Coniacian in age^25^ (Fig. 2). The basin experienced a general shallowing trend from deep lacustrine deposits in Member 1 of ${\text{K}}_{2} {\text{qn}}$^62^, to fairly deep and shallow lake in Members 2 and 3 of ${\text{K}}_{2} {\text{qn}}$^63^.

### Deep Ivorian Basin (DIB)

The DIB belongs to that of Togo-Benin-Nigeria, to a vast sedimentary complex whose subsidence began in the Cretaceous. It is a quasi-covered basin, and situated off equatorial West Africa (Fig. 1); whose formation is related to the expansion of the opening Equatorial Atlantic Gateway^57^. During ODP Leg 159, intended to highlight the process of sedimentation and deformation alongside the Cote d’Ivoire-Ghana Transform Margin, Cretaceous strata were recovered at specific locations 959, 960, 961 and 962 with about 200 m of Upper Cretaceous recovered at site 959D^64^. From bottom to top formations, five lithostratigraphic units subdivide hole 959D^65^ namely Unit V, Unit IV, Unit III, Unit II and Unit I. Relatively well-preserved black shale and siltstones from the Upper Cretaceous to Lower Paleocene (lithologic Unit III) overlie mixed siliceous and carbonate clastics (lithologic unit IV) of the early Coniacian through to the late Albian^65^ (Fig. 2). Deposition of black shales started during the Turonian when the margin differentiation commenced and continuous subsidence generated a semi-enclosed sub-basin^57^, and was controlled by the climate changes in equatorial Africa.

### Demerara rise (DR)

The DR is a submarine plateau off the coast of Suriname and French Guyana on the northern slope of South America^66^ (Fig. 1). Built on rifted continental crust of Precambrian and Mesozoic era, an important part of the submarine plateau is covered by 2–3 km of Cretaceous to Holocene shallow-marine to pelagic sediments. Five key locations were drilled at DR during ODP Leg 207. Site 1261 is located on the northwest flank of the DR approximately 350 km north of Suriname^67^. Five lithostratigraphic units summarize the stratigraphic background at site 1261 whose 89 m of Upper Cenomanian to Lower Santonian sediments represent the lithostratigraphic unit IV, with 650.21–563.3 mbsf sediments recovered at site 1261B^68^. These sediments are mainly constituted of calcareous claystone with organic matter combined with clayey chalk with nannofossils and clayey limestones (Fig. 2). DR experienced a consistent deepening trend during the accumulation of the organic-rich sediments from Cenomanian to early Campanian^69^.

### Results

**Figure 3:**
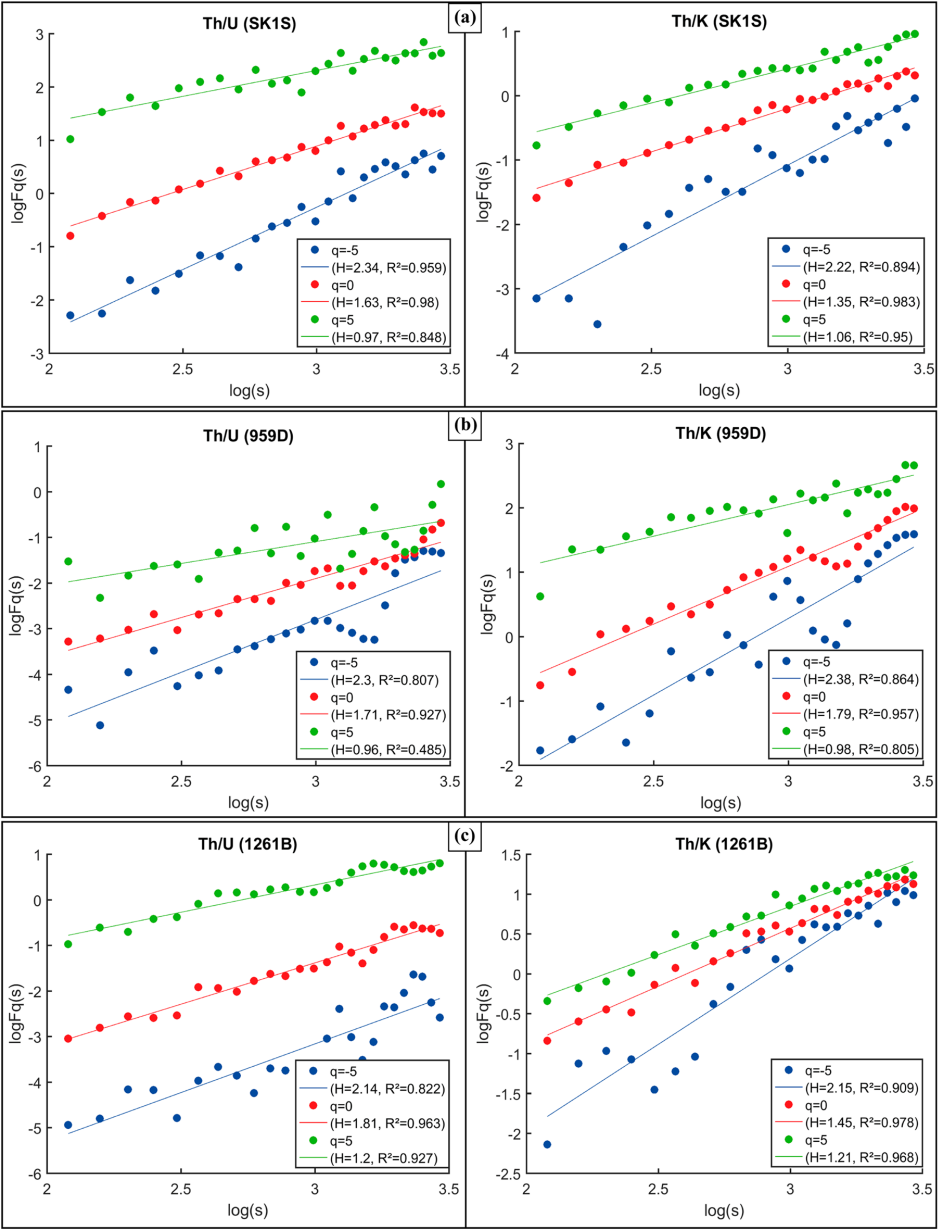
Scaling properties of log–log plots of the fluctuation function (*Fq*(*s*) versus *q*) of Th/U and Th/K ratios in the studied OAE3 units recorded in: (**a**) SLB (borehole south of the Chinese Continental Scientific Drilling Project: SK1S), (**b**) DIB (hole 959D of ODP Leg 159) and (**c**) DR (hole 1261B of leg 207); and their associated regression lines.

**Figure 4:**
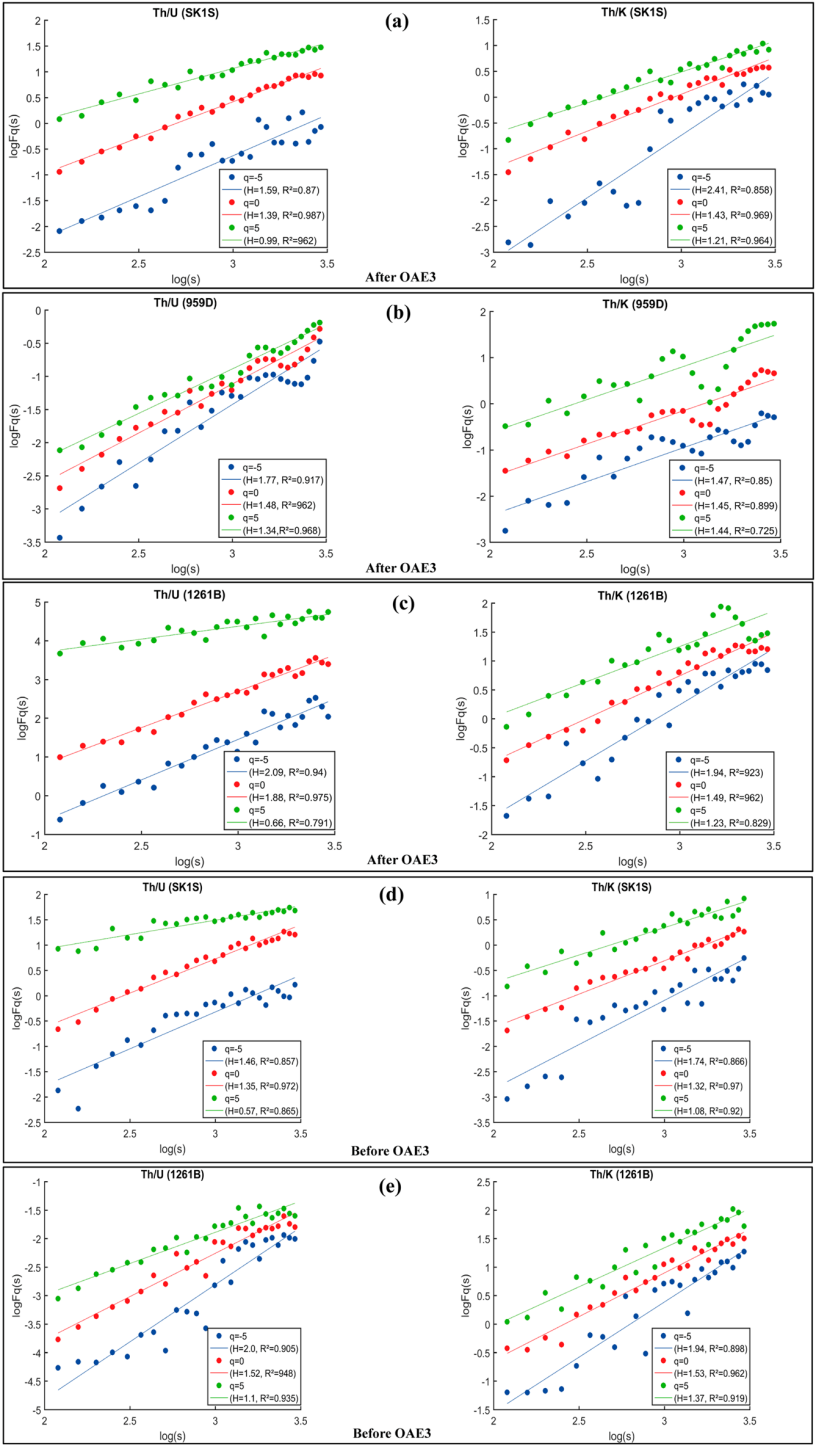
Scaling properties of log–log plots of the fluctuation function (Fq(s) versus q) of Th/U and Th/K ratios before and after the OAE3 interval in: (**a**,**d**) SLB (borehole south of the Chinese Continental Scientific Drilling Project: SK1S), (**b**) DIB (hole 959D of ODP Leg 159) and (**c**,**e**) DR (hole 1261B of leg 207); and their associated regression lines.

The multifractal characteristics of the Th/U and Th/K ratios of the OAE3 and non-OAE sections at SLB, DIB and DR were studied by the MFDFA. Prior to the MFDFA application, the datasets were first segmented and integrated random walk time series into non-overlapping segments with different scaling (except DIB before the OAE3 due to the logging depth). By using Eq. (5) and for − 5 < *q* < 5, the fluctuation function (*Fq(s)* versus *s*) of the Th–K and Th–U distributions have been computed. The scaling behavior obtained by the log–log plots of the fluctuation function of the Th/U and Th/K ratios of the OAE3 and non-OAE units in different boreholes at SLB (SK1S), DIB (959D) and DR (1261B) are shown in Figs. 3 and 4, respectively. All the generalized Hurts exponents (*Hq*) of the Th–K and Th–U distributions in each OAE3 and non-OAE segments are *q* dependent (decrease with the increasing of *q*). The slope of all the fluctuation functions at different scale decrease with the increase of *q*, implying that the Th–U and Th–K distributions of the studied OAE3 and non-OAE intervals have a multifractal property^34,70,71^. By analyzing the slopes, it appears that the scaling properties of the Th–K and Th–U distributions at SLB, DIB and DR are closer in the OAE3 interval than the non-OAE3 intervals (Figs. 3, 4; Table 1). Moreover, the scaling behavior of the Th/U ratio in SK1S and 959D are nearly uniform for all *q* values which may infer some similarities in the thorium and uranium distributions in the OAE3 units in both boreholes (Fig. 3). Also considering the Th/K ratio, some similarities in the scaling properties of the Th/K ratio recorded in the studied OAE3 intervals in SK1S and 1261B can be noticed (Fig. 3).

**Table 1:** Slope difference of the fluctuation functions of the thorium–uranium and thorium–potassium distributions between localities before, within and after the OAE3 intervals.

|$${\Delta H}_{{F_{q} \left( s \right)}}$$|	Th/U			Th/K		
	SK1S-959D	SK1S-1261B	959D-1261B	SK1S-959D	SK1S-1261B	959D-1261B
**After OAE3**						
q = − 5	0.18	0.5	0.32	0.94	0.47	0.47
q = 0	0.09	0.49	0.4	0.02	0.06	0.04
q = 5	0.35	0.33	0.68	0.23	0.02	0.21
**OAE3**						
q = − 5	0.04	0.2	0.16	0.16	0.07	0.23
q = 0	0.08	0.18	0.1	0.44	0.1	0.34
q = 5	0.01	0.23	0.24	0.08	0.15	0.23
**Before OAE3**						
q = − 5	–	0.54	–	–	–	0.2
q = 0	–	0.17	–	–	–	0.21
q = 5	–	0.53	–	–	–	0.29

SK1S (Songliao Basin), 959D (Deep Ivorian Basin), and 1261B (Demerara Rise).

**Figure 5:**
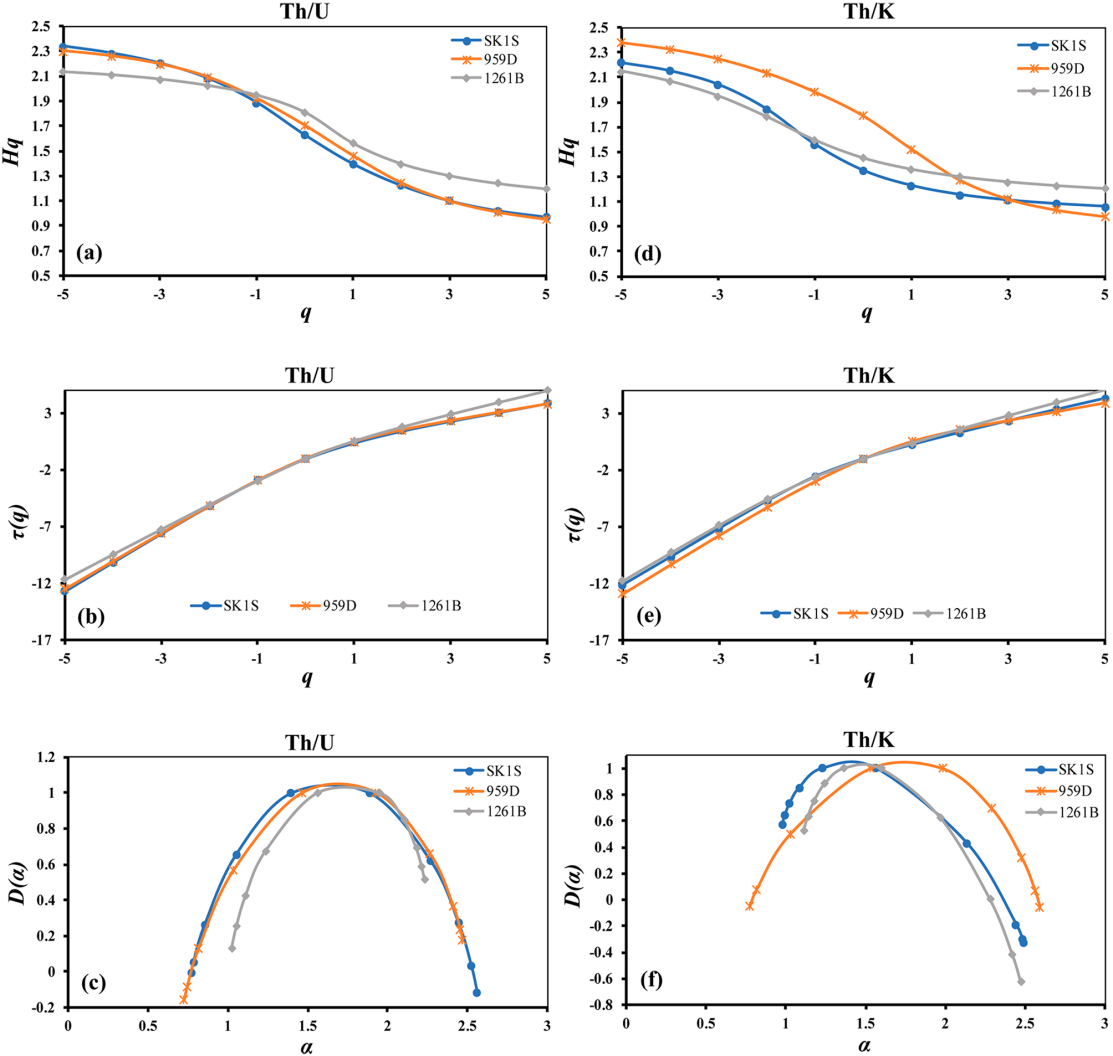
The Multifractal properties of the thorium–potassium and thorium–uranium distributions in the studied OAE3 intervals recorded in Songliao Basin (SK1S), Deep Ivorian Basin (959D) and Demerara Rise (1261B). (**a**) The generalized Hurst exponents of the Th/U ratio. (**b**) The mass exponents of the Th/U ratio. (**c**) The multifractal spectrum of the Th/U ratio. (**d**) The generalized Hurst exponents of the Th/K ratio. (**e**) The mass exponent of the Th/K ratio. (**f**) The multifractal spectrum of the Th/K ratio.

**Figure 6:**
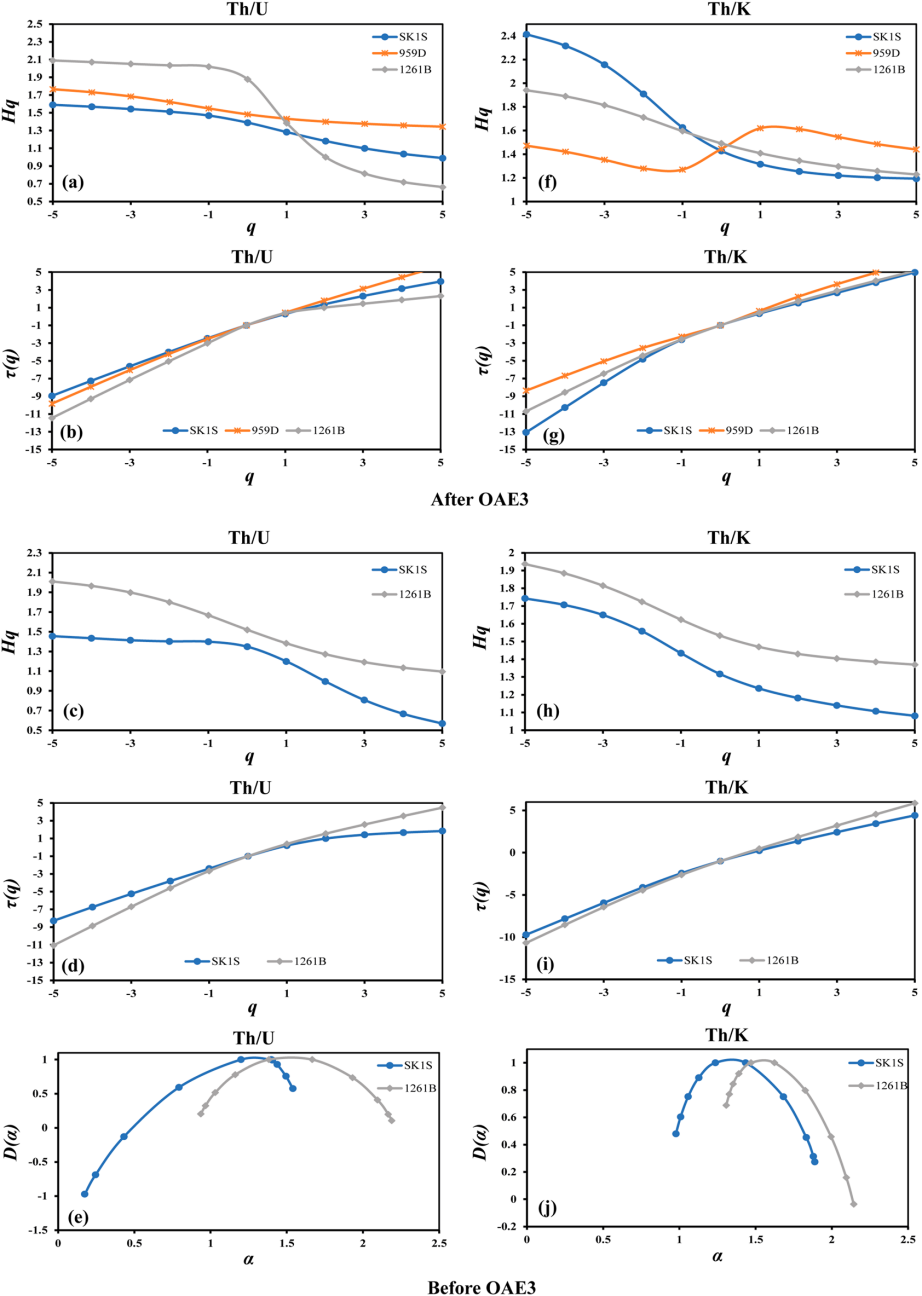
The Multifractal properties of the thorium–potassium and thorium–uranium distributions before and after the OAE3 intervals recorded in Songliao Basin (SK1S), Deep Ivorian Basin (959D) and Demerara Rise (1261B). *Hq*, the generalized Hurst exponents. *τ(q)* the mass exponents, *D(α)* the multifractal spectrum.

Besides the fluctuation function, the multifractal properties of the studied OAE3 (Fig. 5) and non-OAE3 (Fig. 6) segments in SLB, DIB and DR were verified by determining the generalized Hurst exponent *h*(*q*), the mass exponent *τ*(*q*) and the multifractal spectrum *D*(*α*) using Eqs. (7), (8) and (9), respectively. The generalized Hurst exponents *h*(*q*) vary with *q* of the Th/U and Th/K ratios in the three different boreholes (Figs. 5a,d, 6a,c,f,h), implying the multifractal behavior (except Th–K distribution at DIB after the OAE3) as described by the scaling properties. In the OAE3 segment, the *q*-dependence of *h*(*q*) of the Th/U ratio in SK1S and that of 959D are quasi-similar, but such similarity was not observed in the non-OAE units. Furthermore, by considering the mass exponent derived from the fluctuation function (Figs. 5b,e, 6b,d,i,g), the Th/U and Th/K ratios of all the studied OAE3 and non-OAE3 units are multifractal in nature (except Th–K distribution at DIB after the OAE3) since the mass exponent plots show a nonlinear *τ*(*q*) dependence on *q*^72^. The degree of multifractality can be evaluated by the degree of the nonlinearity of the mass exponent for *q* < 0 and *q* > 0^42,72^. Table 2 shows that on average, the highest slope differences of the mass exponent *τ*(*q*) between the negative (*q* < 0) and positive (*q* > 0) *q*-order of Th/U and Th/K ratios are found in the OAE3 interval, indicating that the Th–U and Th–K distributions in this depositional interval possess a higher degree of multifractality in their scaling properties. In addition, the slope difference of Th/U and Th/K ratios in SK1S and 959D are close within this unit, which may infer some similarities in their degree of multifractality.

**Table 2:** Slope values of mass exponent *τ*(*q*) for thorium–uranium and thorium–potassium distributions before, within and the OAE3 intervals at Songliao Basin (SK1S), Deep Ivorian Basin (959D) and Demerara Rise (1261B).

Sites	Th/U			Th/K		
	SK1S	959D	1261B	SK1S	959D	1261B
**After OAE3**						
*q* ∈ [ − 5 ; 0 [	1.62	1.82	2.11	2.63	1.53	2.04
*q* ∈ ] 0 ; 5 ]	0.91	1.32	0.47	1.16	1.39	1.18
**OAE3**						
*q* ∈ [ − 5 ; 0 [	2.46	2.41	2.19	2.4	2.49	2.3
*q* ∈ ] 0 ; 5 ]	0.86	0.82	1.1	1.02	0.84	1.17
**Before OAE3**						
*q* ∈ [ − 5 ; 0 [	1.47	–	2.1	1.83	–	2.02
*q* ∈ ] 0 ; 5 ]	0.4	–	1.02	1.04	–	1.34

The singularity spectrum *D*(*α*) and the singularity exponent *α* describing the multifractal spectrum of the Th/U and Th/K ratios of the different formations in the OAE3 and non-OAE3 units are displayed in Figs. 5c,f and 6,e,j, respectively. The curves described by all the multifractal spectrums are asymmetric unimodal with different singularity spectrum widths, implying that under fluctuating environmental settings, the Th–K and Th–U distributions in the studied OAE3 and non-OAE3 intervals present varying multifractal characteristics. Furthermore, the singularity spectrum widths defined by Δ*α* = $\alpha_{max}$ − $\alpha_{min}$, which reflect the degree of multifractality/complexity were calculated using Eq. (11) (Table 3). When Δ*α* is low, the whole system is described to possess low heterogeneity in its local scaling and vice versa^73^. Thus, the results show that on average, the OAE3 interval has the larger multifractal spectrum widths of both Th/U and Th/K ratios, which may indicate that the temporal fluctuation of both Th–U and Th–K distributions in this unit display a high degree of multifractality/complexity, with a more complex trend in SK1S for Th–U distribution and a more complex trend in 959D for Th–K distribution.

**Table 3:** The multifractal spectrum widths of the thorium–uranium and thorium–potassium distributions before, within and after the OAE3 intervals at Songliao Basin (SK1S), Deep Ivorian Basin (959D) and Demerara Rise (1261B).

Δ*α *= ${\varvec{\alpha}}_{{{\varvec{max}}}}$ − ${\varvec{\alpha}}_{{{\varvec{min}}}}$	Th/U			Th/K		
	SK1S	959D	1261B	SK1S	959D	1261B
**After OAE3**						
Original data	0.88	0.62	1.73	1.65	0.42	1.03
Shuffled	0.72	0.18	1.76	0.39	0.52	0.4
Surrogate	1.9	0.24	1.45	1.11	0.39	0.98
**OAE3**						
Original data	1.79	1.74	1.21	1.51	1.82	1.37
Shuffled	0.87	0.91	0.37	0.34	0.56	0.52
Surrogate	0.95	0.97	0.75	1.3	1.01	0.61
**Before OAE3**						
Original data	1.37	–	1.25	0.91	–	0.84
Shuffled	0.69	–	0.53	0.29	–	0.75
Surrogate	1.95	–	0.98	0.81	–	0.88

## Discussion

### Sources of multifractality

In general, the multifractality observed in time or spatial series have two main causes^38,42,43,73^: (**1**) the multifractality originating from a wideness of the probability density function (PDF), and (**2**) the multifractality caused by long-range correlation for small and large fluctuations in the pattern arrangement. To highlight the dominant one between these two types of multifractality in the Th–K and Th–U distributions in the studied OAE3 and non-OAE3 intervals, we analyzed the corresponding randomly shuffled and surrogate datasets generated using the original Th–K and Th–U datasets based on Kimiagar et al.^41^, Movahed et al.^39^ and Niu et al.^40^. Indeed, the shuffled process removes the multifractality due to long-range correlation while retaining the multifractality caused by the broad PDF signal. Thus, the shuffled dataset will exhibit non-multifractal behavior, with *h*(*2*) = 0.5 if the multifractality properties are solely controlled by the long-range correlation. In case the multifractality of the analyzed Th–K and Th–U datasets originates from both types of multifractality, the shuffled series will show weaker multifractality than the original series^39^. By considering the surrogate method, the generalized Hurst exponents *h*(*q*) generated have the capability to be *q*-independent if the multifractality observed in the Th–K and Th–U distributions is mainly from the wideness of PDF. However, if the multifractality properties are triggered by both sources, then weaker multifractality would be found in both shuffled and surrogate data of the Th–K and Th–U datasets^39,42^.

**Figure 7:**
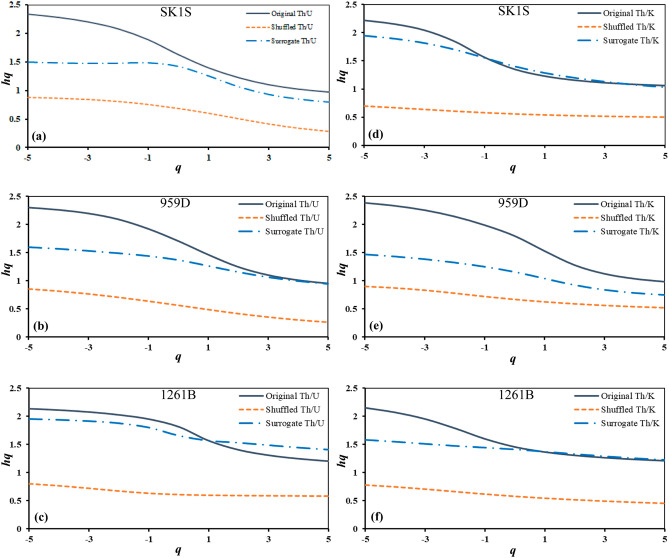
The generalized Hurst exponent of shuffled and surrogate data series computed based on the original thorium–potassium and thorium–uranium distributions in the studied OAE3 intervals recorded in Songliao Basin (SK1S): (**a**,**d**); Deep Ivorian Basin (959D): (**b**,**e**); Demerara Rise (1261B): (**c**,**f**).

Hence, to explore the origins of multifractality in boreholes SK1S, 959D and 1261B, firstly, the shuffled and surrogate procedure were applied to the original Th–K and Th–U datasets of the OAE3 interval, and the results are shown in Fig. 7. Afterwards, the same procedure was applied to the non-OAE3 units (Table 3).

From Fig. 7, it is observed that the generalized Hurst exponents *h*(*q*) of the shuffled data set are slightly *q* dependent for all Th/U and Th/K ratios with monotonically decreasing curves. Also, the analysis of the generalized Hurst exponents indicates that for *q* = *2*, *h*(*q*) slightly converge to 0.5. This trend shows that the multifractal behavior described in the Th/U and Th/K ratios is due to the broad PDF and the long-range correlation. However, by analyzing the surrogate curves, we observe that the generalized Hurst exponents *h*(*q*) of the surrogate series are all quasi *q*-dependent, suggesting that the multifractality due to long-range correlation is dominant in the Th–K and Th–U distributions in the OAE3 intervals. Tanna and Pathak^74^, and Wu et al.^42^ described a similar trend where both the broad PDF and the long-range correlation were the cause of the multifractality when studying an ionospheric scintillation time series and hydrological data, respectively. To identify the influence of the broadness of the PDF and the long-range correlation in their dataset, the dimension of the multifractal spectrum width of the original time series was correlated to the estimated dimensions of the multifractal spectrum width of the shuffled and surrogate time series. Their results indicated a narrow multifractal spectrum width of the shuffled time series compared to that of the surrogate time series, which also had a narrow multifractal spectrum width compared to that of the initial dataset. Subsequently, they concluded that the contribution of the broad PDF on the multifractal behavior observed in their dataset was weaker than the long-range correlation.

Following the same procedure, the multifractal spectrum widths of the original, shuffled and surrogate series of the Th–U and Th–K distributions were examined for the OAE3 interval as well as the non-OAE3 units (Table 3). Through Table 3, it is obvious that the multifractality triggered by the long-range correlation is dominant in the OAE3 intervals in all the boreholes, while it is not the case in the non-OAE3 units. In SK1S, the $\Delta \alpha_{Surrogate}$ of Th–U distribution is stronger than $\Delta \alpha_{Original}$ before and after the OAE3 interval, indicating that the PDF dominates the multifractality^75^. Th–K distribution exhibits similar behavior before the OAE3 interval at site 1261B. Moreover the multifractal behavior of the Th–U and Th–K distributions after the OAE3 intervals at sites 1261B and 959D, respectively is dominated by the PDF since $\Delta \alpha_{Shuffled}$ is stronger than $\Delta \alpha_{Surrogate}$^42^. Thus, the main cause of the multifractal behavior of the Th–U and Th–K distributions in the non-OAE3 intervals is non-homogeneous, however, it is homogeneous in the OAE3 interval (Table 3). Consequently, is there a common factor controlling the multifractality in the OAE3 intervals in the three basins?

### Possible factors affecting the multifractal properties of Th–U and Th–K distributions in the OAE3 interval

The Th/U and Th/K ratios in sediments fluctuate under a number of different controls such as clay mineral content, paleo-redox conditions and paleoclimate^53,76^.

Th/K ratio is for the most part a function of clay mineral content in shale formations^77^. Previous studies have shown that the presence of shale and variations in the sub-surface sedimentation pattern largely influence the multifractal behavior of the gamma ray log response^44,45^.

**Figure 8:**
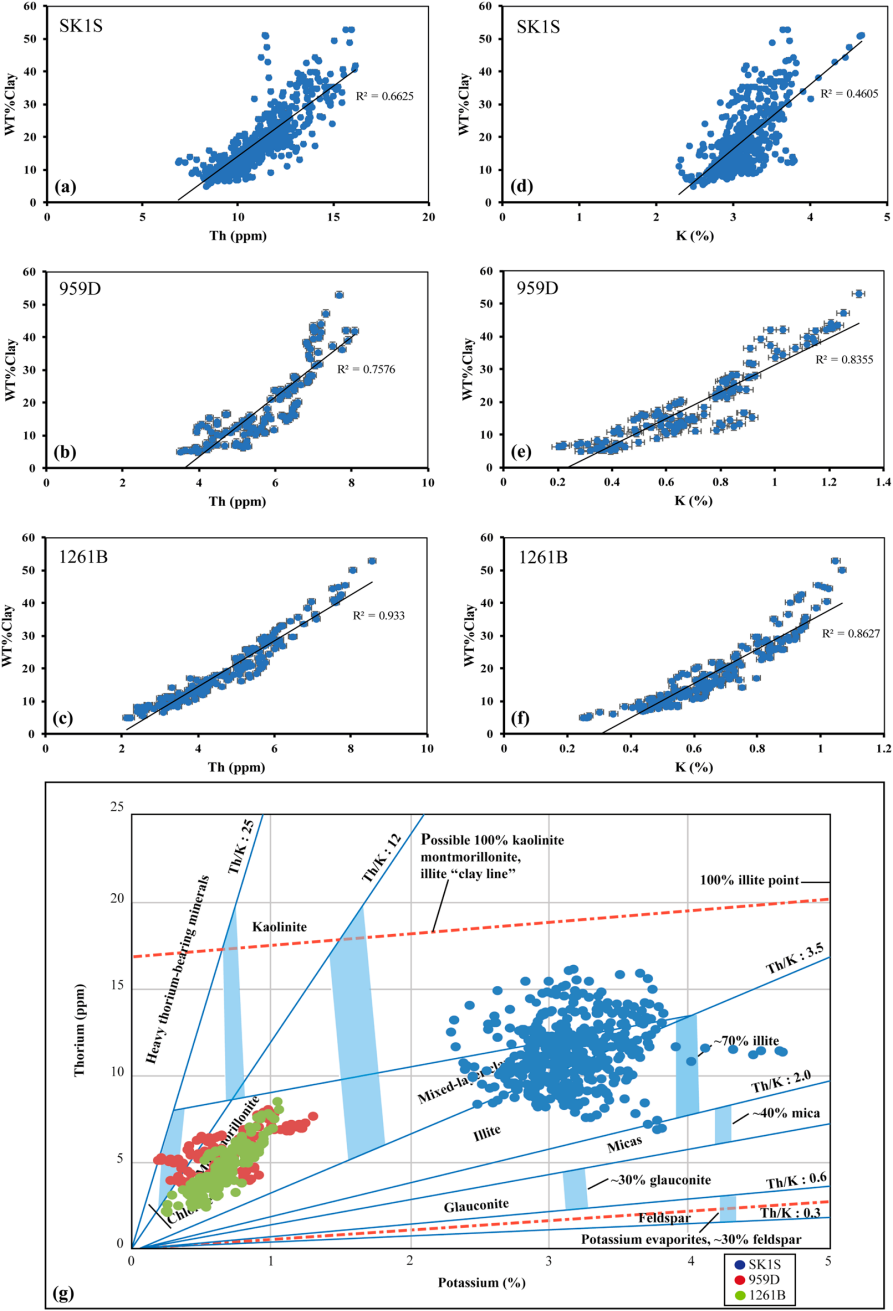
Correlation between the weight percent of clay, Th and K contents; and Clay minerals evaluation in the OAE3 sections recorded in Songliao Basin, Deep Ivorian Basin and Demerara Rise based on Schlumberger reference thorium–potassium cross-plot (log interpretation charts 2013).

Based on the thorium and potassium distributions, we analyzed the likely relationship between clay minerals and studied OAE3 intervals multifractality (complexity) in each borehole through a Th–K cross-plot (Fig. 8g) and singularity spectrum width correlation (Table 2). However, since thorium and potassium can also be associated with non-clay minerals, we first evaluated the correlation between Th and K, and the estimated weight percent of clay (WT% clay) based on the empirical expression developed by Bhuyan and Passey^78^ (Fig. 8a–f). WT% clay represents the total clay obtained from the total GR. Meanwhile, the dispersions between the total GR and Th, K and U elements are useful to investigate the likely sources of these elements^79^. Therefore following Ito et al.^79^, a strong correlation between WT% clay, and Th and K suggests that clay is the main contributor of Th and K responses, while weak correlation suggests that the main source of Th and K may not be the clay minerals. From Fig. 8a–f, a good positive correlation appears between the weight percent of clay, and Th and K variations at sites 959D and 1261B, while in SK1S, the correlation is moderate for K. The analysis of the correlation coefficients supposes that clay minerals are the predominant contributors of Th and K responses, and therefore led to clay type evaluation by a Th–K cross-plot (Fig. 8g). The results show that in the three boreholes, the nature of dominating clay minerals are different just as it differs from the singularity spectrum widths Δ*α*. In 1261B, the OAE3 segment is dominated by a single type of clay mineral mainly montmorillonite (smectite) and the estimated dimension of the singularity spectrum width is the lowest (Δ*α* = 1.37). In borehole 959D, three types of clay minerals (montmorillonite, chlorite heavy thorium bearing minerals) dominate the OAE3 unit and the estimated dimension of the singularity spectrum width is the highest (Δ*α* = 1.82) (Table 3). At SK1S, although the TEOAE3 interval is dominated by three categories of clay minerals i.e. mixed layer clays (illite-montmorillonite mainly), illite and micas (Fig. 8g), the estimated dimension of the spectrum width is less than that of 959D (Δ*α* = 1.51) (Table 3). This partially falls in line with the findings of Gao et al.^80^, where similar clay distribution results with illite, illite–smectite and chlorite were found by studying SK1S clay minerals distribution.

These results suggest that the multifractal behavior of the Th–K distribution in the studied OAE3 intervals is influenced by the nature and diversity of clay minerals, and changes in the deposit environment. This result is consistent with the work of Dashtian^44^. Looking at the above differences of thorium and potassium distributions in these three OAE3 intervals, it suggests that the main factors controlling their scaling behavior during the development of the Coniacian–Santonian OAE are different. Therefore, the long-term persistence of the Th–K distribution might be related to the persistence in the deposition of clay minerals and/or Th–K rich minerals under different sedimentation patterns. Furthermore, the change in the shape of the singularity spectrum for Th/K and Th/U ratios may indicate some variations in the thorium–potassium and thorium–uranium distributions. When the multifractality is sensitive to the local fluctuations with small amplitudes, the singularity spectrum will be found with left side shortness^74^. In all boreholes, the left side shortened spectrum of the Th/K ratio of the studied OAE3 intervals (Fig. 5f) suggests the presence of local changes with small amplitudes in the depositing of the clay minerals. Sedimentary clay minerals derive from the rock weathering in the source area, and are influenced by provenance lithology, paleoclimate, depositional environments and rocks formation (diagenesis)^81–83^.

In borehole 959D of the DIB, Kennedy and Wagner^84^ linked the clay mineral properties change observed in the Coniacian–Santonian black shales to the faint precession-driven fluctuations of continental climate. They theorized that periodical shift of the intertropical convergence zone controlled by minor orbital-driven changes triggered an increasing rainfall and weathering leading to the genesis of smectite-rich clay^84^. This climatic control is supported by the works of Chamley^81^, who correlated the kaolinite abundance found in the equatorial Eastern Atlantic Ocean to a strong climatic control regulated by the intensity of the continental hydrolysis. Furthermore, the left-side truncated spectrum of Th/K ratio (Fig. 5f) indicates the existence of local intermittency in the thorium and potassium deposition, which may suggest the existence of local fluctuations in the climate evolution. Previous studies based on climate tracers at site 959 showed the presence of highly fluctuating climate modulated at different timescales during the Upper Cretaceous^54^. Thus, since the Th/K ratio depends on the nature of the clay mineral assemblage, which are controlled by climatic fluctuations, we correlate the change in the multifractal features of the Th–K distribution to the change in the climate dynamics during the Coniacian–Santonian OAE3 in the DIB, since several scholars have shown that the multifractality properties of geological data change with the changes in the climatic processes^73,85–87^.

At DR (borehole 1261B), the Late Cretaceous clastic sediments derive from weathered granitoid basement rocks of the Guyana Shield^67,88^. These sediments afterwards were accumulated under seasonal and long-term (mainly eccentricity and obliquity) bands^89^. The existence of smectite (montmorillonite) as the predominant type of clay mineral in the Coniacian–Santonian OAE3 interval (Fig. 8g) suggests an increase in chemical weathering^81^ under small seasonal climate fluctuation during its depositional time, because smectite is known as a product derived from a seasonal climate, minor changes in continental climate^84^. Based on carbonate analysis recorded alongside DR, Nederbragt et al.^90^ also reported a tropical seasonality on DR throughout the Turonian to Santonian. The local intermittency in the thorium and potassium deposition revealed by the multifractal spectrum width of the Th/K ratio may also be due to this seasonal change. Therefore, the abundance of one type of clay mineral implies that the seasonal fluctuation is far from the main factor controlling the thorium–potassium ratio. Consequently, the multifractal nature of the thorium–potassium distribution observed in the OAE3 segment in borehole 1261B may be due to persistence in Al-rich weathered granitoid basement rocks from the Guyana Shield resulting from minor seasonal climate fluctuations chiefly controlled by eccentricity and obliquity.

**Figure 9:**
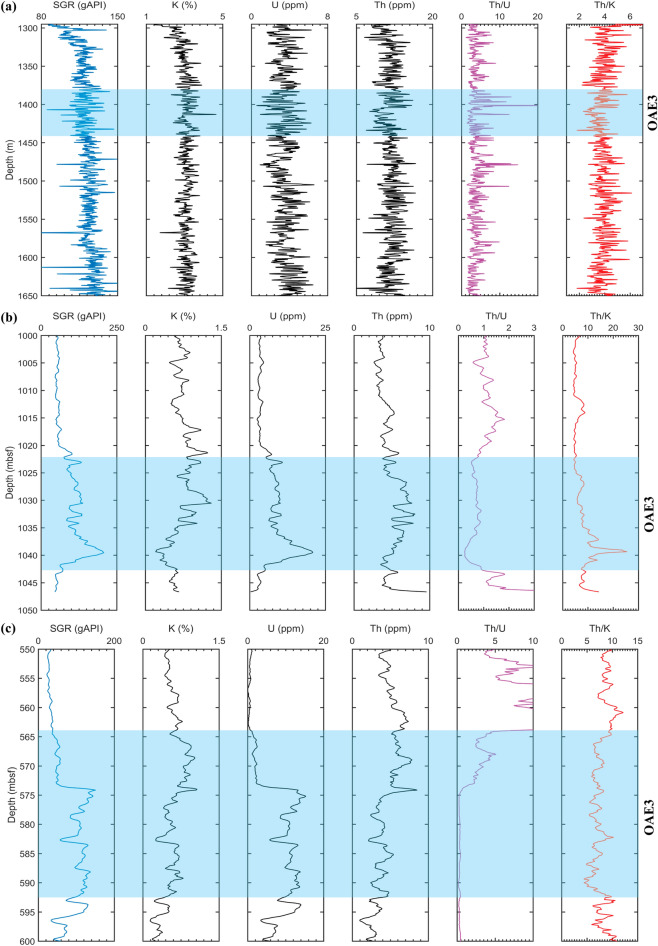
Spectral gamma ray, the computed Th/U and Th/K ratios, and the studied Coniacian–Santonian OAE intervals in SK1S (**a**), 959D (**b**) and 1261B (**c**). The OAE3 intervals in SK1S, DIB and DR are from Jones et al.^23^, Wegner et al.^98^ and Beckmann et al.^20^, respectively.

Figure 8g shows that illite, mixed layer clays (illite–montmorillonite) and micas dominate the TEOAE3 unit in SK1S without smectite. This trend was described by Gao et al.^80^ as a consequence of burial diagenesis due to the fact that illite persists during burial diagenesis, while smectite is transformed in illitic layers—associated with geochemical changes involving K and Al dissolved from proximate feldspars and mica^91^. Since SLB is enclosed by crystalline igneous outcrops^92^, their weathering would have released Al and K which get integrated in the smectite layers to form illitic clays. This is consistent with the medium correlation between the weight percent of clay and K in SK1S (Fig. 8d) indicating that a substantial part of K may derive from non-clay minerals. Furthermore, based on climatically sensitive proxies, Wang et al.^26^ found that SLB did not undergo large climatic variations throughout the Late Cretaceous but experienced abundant rainfall inferring that local fluctuations were common phenomena in the paleo-basin. This seems consistent with the left-side truncated spectrum of Th/K ratio (Fig. 5f) indicating the existence of local intermittency in the thorium and potassium deposition, which may suggest the existence of local fluctuations during the OAE3. Since, the burial diagenesis is the main process controlling the clay mineralogical change below 1,100 m in SK1S^80^, and the basin did not experience large climatic shifts^26^, and as the TEOAE3 interval in SLB ranges from 1,380 to 1,440 m (Fig. 9a), we therefore correlate the multifractality of the lacustrine OAE3 Th/K ratio to a long-term persistence of the local burial diagenesis during the basin subsidence under the control of local tectonic and climatic variations^80,93^, which played an important role during the basin structuration.

**Figure 10:**
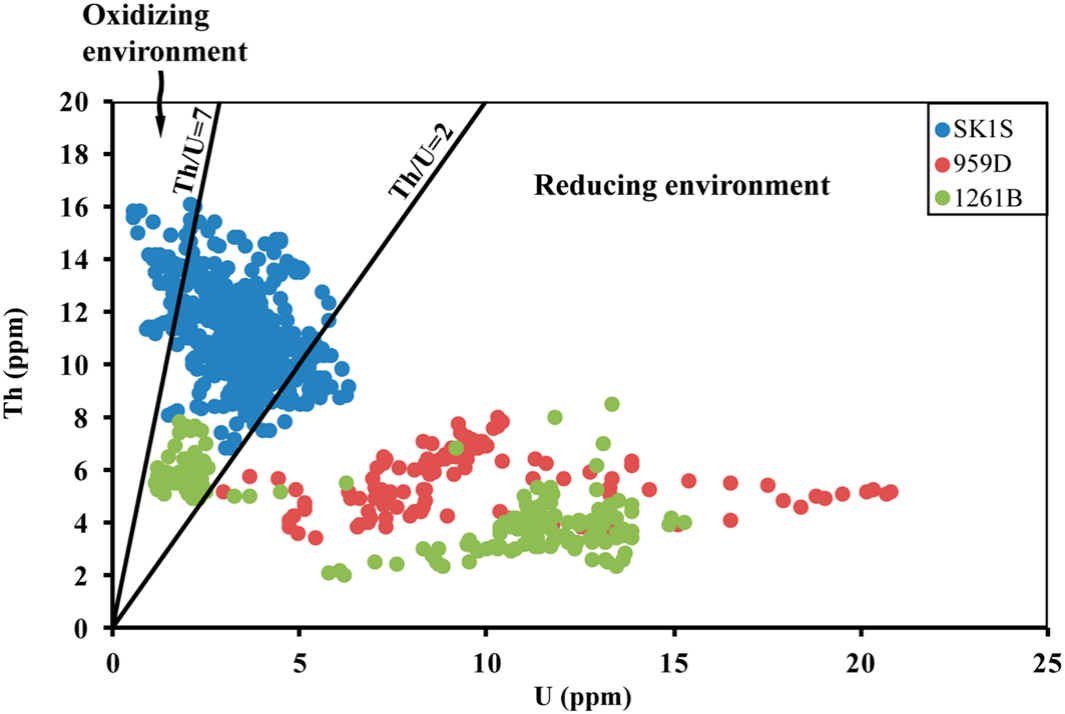
OAE3 paleo-redox conditions characterization in Songliao Basin, Deep Ivorian Basin and Demerara Rise based on thorium–uranium cross-plot. Th/U < 2 represents a reducing environment; Th/U > 7 represents an oxidizing environment; 2 < Th/U < 7 represents a transitional period between oxidizing and reducing environment.

Similar to the Th/K ratio, the probable factors affecting the multifractal properties of the thorium–uranium distribution in the studied OAE3 intervals were analyzed in all boreholes by correlating the thorium–uranium cross-plot (Fig. 10) and the singularity spectrum results. Th/U ratio is a useful proxy to track the paleo-redox conditions of the original sedimentary environment and/or subsequent diagenetic processes^94^ since it is often strongly connected with the changes in the depositional environment^95^. The depositional environment is probably described as reducing when Th/U < 2 (commonly marine), or oxidizing when Th/U > 7 (possibly terrestrial)^95,96^. Based on Fig. 10, the three OAE3 intervals were deposited in different paleo-redox conditions. In boreholes 959D and 1261B, the Th/U ratio shows that the OAE3 unit is mainly characterized by reducing environment while in SK1S it is characterized by a period ranging from oxidation to reduction sedimentation.

According to San José Martínez et al.^97^ and Liu et al.^34^, the shape features of the multifractal spectrum defined by Δ*D* = $D\left( {\alpha_{max} } \right)$ − $D\left( {\alpha_{min} } \right)$ can estimate the probability of the dominate subset in time series. When Δ*D* < 0, a small probability subset dominates, while a large probability subset dominates when Δ*D* > 0. Therefore, a period of strong and long-term reducing environment dominates in the OAE3 interval at sites 959D and 1261B and correlates well with Δ*D* > 0 (Table 4). This is supported by the computed average of Th/U ratio in both boreholes ((Th/U)_mean_ = 0.66 and (Th/U)_mean_ = 1.3, respectively). Previous studies based on geochemical data analysis found a similar result at both sites^17,57,67,89^. Furthermore, the analysis of the singularity spectrum of the Th/U ratio indicates a right-side shortness of the multifractal spectrum (Fig. 5c), reflecting the existence of small-scale intermittency in the thorium–uranium distribution at both sites^74^, which may reveal some shifts in the redox conditions during the deposition of the OAE3 interval. This result is consistent with the works of März et al.^17^ and Hofmann and Wagner^67^ who described periodic changes between anoxic and euxinic bottom water condition under periodic shifts of the Intertropical Convergence Zone during the deposition of the Coniacian–Santonian OAE3 intervals at DR and DIB. Hence, the multifractal nature of the thorium–uranium distribution in the OAE3 intervals may be due to long-term persistence of reducing conditions at sites 959D^98^ and 1261B^55^, which contributed to the deposition of black shale at DR and DIB.

**Table 4:** The multifractal spectrum shape features of the thorium–uranium distribution in the studied OAE3 intervals.

Multifractal spectrum shape	Th/U		
	SK1S	959D	1261B
Δ*D* =$D\left( {\alpha_{\max } } \right)$ − $D\left( {\alpha_{\min } } \right)$	− 0.108	0.336	0.384

In SK1S, Δ*D* < 0 (Table 4) suggesting that there is a small probability that neither the oxidizing nor reducing environment dominates during the OAE3. This result is consistent with the computed mean of Th/U ratio ((Th/U)_mean_ = 4.04) in the TEOAE3 interval, indicating that the OAE3 occurred during a transitional period between reducing and oxidizing sedimentation as also revealed by Jones et al.^23^. As mentioned before, the multifractal singularity spectrum displays left-side shortness when the dataset has a multifractality that is sensitive to the local fluctuations with minor amplitudes. In the case of SK1S, the thorium–uranium distribution suggests the presence of local fluctuations under minor changes since the multifractal spectrum of the Th/U ratio exhibits left-side shortness (Fig. 5c). The multifractal behavior of the thorium–uranium distribution may be a consequence of long-term persistence of local changes like increased seasonality, freshening of bottom-water, and/or lake depth reducing which likely contributed to the reoxygenation of the water column and consequently, the demise of the reducing condition^23^.

The computed multifractal spectrum widths of Th/U ratio differ from OAE3 segment to OAE3 segment indicating distinct redox-potential strength (Table 3). The expression of the OAE3 unit recorded in SK1S displays the highest singularity spectrum width of Th/U ratio implying that this interval was deposited in more complex paleo-redox environment compared to those in boreholes 959D and 1261B, as also revealed by the thorium–uranium cross-plot (Fig. 10) and by the fractal dimensions (Table 5) estimated using the commonly used box dimension (D_B_)^99^ and Higuchi dimension (D_H_)^100^ defined by:

1 $$ D_{B} = \mathop {\lim }\limits_{\delta \to 0} \frac{{\log N_{\delta } }}{ - \log \delta }\;{\text{and }}D_{H} = - \frac{{d\ln \left( {L\left( k \right)} \right)}}{d\ln \left( k \right)} $$

where *N* is the number of thorium–uranium data series and $\delta$ the scale; *L*(*k*) the normalized length of the data series.

**Table 5:** Estimated fractal dimensions of thorium–uranium distribution in the studied OAE3 intervals.

Locations	Box dimension	Higuchi dimension
SK1S	1.64	1.89
959D	1.58	1.54
1261B	1.46	1.31

We hypothesize that the changes in the paleo-redox conditions influenced the multifractal properties of the thorium–uranium distribution in the studied OAE3 intervals.

**Figure 11:**
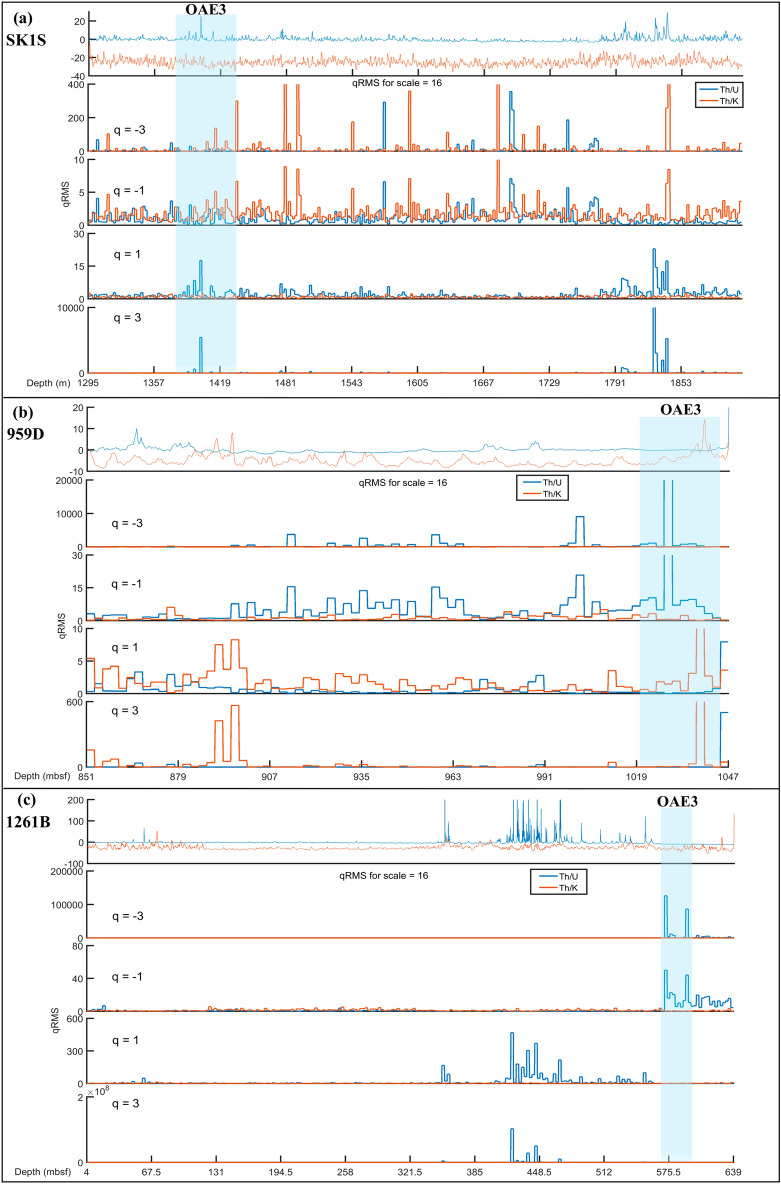
The *q*-order RMS of the thorium–uranium and thorium–potassium distribution in the studied OAE3 intervals.

Furthermore, the similarities of the paleo-redox conditions during the OAE3 were explored through the root-mean-square (RMS) variation which helps to study the average variation of time series. Indeed, the RMS can be used to segregate the fluctuation function between the amplitudes of the local changes^71^ based on the *q*-order variation. The scaling properties of RMS quantifies the variation of the fluctuation function over the interval with large and minor changes. In marine environment (959D and 1261B), the fluctuation function of thorium–uranium distribution is characterized by a period with small variation for negative scaling (*q* < 0) during the OAE3 (Fig. 11a,b), while in the non-marine SLB (SK1S), the fluctuation function of thorium–uranium distribution displays a period with large variation over positive scaling (q > 0) during the OAE3 (Fig. 11c). Therefore, the paleo-redox condition in SK1S differ significantly from that of 959D and 1261B.

Since the OAE3 is known as associated with a period of long-term significant change in the Earth's climate dynamism with a major cooling trend spanning from the mid-Cretaceous mega-greenhouse to the normal greenhouse state^10^, this transition likely triggered changes in the depositional systems. Furthermore, astronomical forcing seems to control the black shale deposition in the OAE3 interval at DIB^21,54^, DR^101^ as well as the depositional processes in SK1S^25^. Thus, in light of the aforementioned detailed discussion, we surmise that the climatic and environmental disturbances during the OAE3 (probably due to an orbital forcing) induced changes in both the weathering mechanism and the deep-water sediments supply which in turn influenced the clay mineralogy and the bottom water redox conditions in marine and continental basins—and consequently the scaling behavior of Th–K and Th–U distributions.

### Conclusions

Based on spectral gamma ray log data recorded in the non-marine Cretaceous Songliao Basin and the marines Deep Ivorian Basin and Demerara Rise, for the first time, the multifractality of the Coniacian–Santonian oceanic anoxic event was explored through analysis of the multifractal behavior of thorium–potassium and thorium–uranium distributions both in marine and terrestrial environments using the MFDFA. The following conclusions can be drawn:

1. The *q*-dependence of the generalized Hurst exponents *h*(*q*), classical scaling exponents and multispectral spectrum indicate that the thorium–potassium and thorium–uranium distribution in the OAE3 interval from both marine and terrestrial records exhibit a multifractal nature. The results of the estimated shuffled and surrogate data series show that the multifractality due to long-range correlation is dominant in the Coniacian–Santonian OAE interval in both marine and terrestrial environments.
2. The multifractal behavior of the studied OAE3 intervals is associated with the presence of clay minerals and change in the paleo-redox conditions. At all sites the left side shortened spectrum of the Th/K ratio estimated based on the spectral gamma ray indicate the presence of local changes with small amplitudes in the deposition of clay minerals during the OAE3. Moreover, the shortened singularity spectrum of thorium–uranium distribution reflects the existence of small-scale fluctuation with large amplitudes in the redox conditions at the marine sites (DIB and DR). Whereas in the Songliao Basin, the singularity spectrum indicates the presence of local fluctuations with small amplitudes during the OAE3.
3. The multifractal features, the fractal dimensions and the RMS of the described Coniacian–Santonian oceanic anoxic events based on the spectral gamma ray differ from each other and correlate with the changes in the sedimentation pattern under different paleoenvironmental conditions in both marine and terrestrial environments, suggesting a more local behavior of the event even though the regional character is not neglected.

## Data and analysis method

### Data

The log gamma records the total natural gamma radiation while the spectral gamma ray log not only indicates the whole intensity of radioactivity, but also estimates quantitively the content of the main radioactive constituents (Th, U and K) in the formation. Because the concentration of natural radioactive elements is mainly a function of the depositional environment and diagenesis^102^, the spectral gamma ray log have been widely used for paleoenvironmental research^51–53^.

The data used in this work are chiefly the spectral gamma ray logs data (Fig. 9) originating from downhole logging using the gamma ray spectrometry tool during the first stage of the Continental Scientific Drilling Project of Cretaceous Songliao Basin (SK1), Ocean Drilling Program (ODP) Leg 159 and ODP leg 207. The Th/K and Th/U ratios were subsequently computed prior to the multifractal analysis due to the fact that these two proxies are sensitive to paleoenvironmental changes.

### Analysis method

The approach applied for the analysis of the spectral gamma ray data in the present paper is the MFDFA. MFDFA has been widely employed to explore the self-similarity nature of nonstationary time series^42,45,73,103,104^. The analysis procedure can be found as follows^38^:

- Determine the fluctuation profile *Y*(t) of the Th–K and Th–U distributions considering $x_{t}$ the data of Th–K and Th–U ratios of length *N*
  2 $$ Y\left( t \right) = \mathop \sum \limits_{i = 1}^{N} (x_{t} - \overline{x}); t = 1, 2, \ldots , N $$
  where $\overline{x}$ is the mean of the data series $x_{t}$.
- Segregate the *N*-length *Y*(t) of the Th–K and Th–U distributions obtained from Eq. (2) into uniform non-overlapping intervals $N_{s} = int\left( {N/s} \right)$ of length *s*. Mostly, because *N* is not a common multiple of *s*, a short part of the fluctuation profile may be left at the end of the division. To have a complete sampling of the whole segment, the segmentation is repeated backwards i.e. starting from the opposite end of the data series. This process provides $2N_{s}$ non-overlapping segments of Th–K and Th–U distributions at the end.
- Estimate the variance of the $2N_{s}$ segments of the Th–K and Th–U distributions by statistical approach:
  3 $$ F^{2} \left( {s, v} \right) = \frac{1}{s}\mathop \sum \limits_{i = 1}^{s} \left\{ {Y\left[ {\left( {v - 1} \right)s + i} \right] - y_{v} \left( i \right)} \right\}^{2} , v = 1, 2, \ldots , N_{s} $$
  4 $$ F^{2} \left( {s, v} \right) = \frac{1}{s}\mathop \sum \limits_{i = 1}^{s} \left\{ {Y\left[ {\left( {N - (v - N_{s} } \right)s + i} \right] - y_{v} \left( i \right)} \right\}^{2} , v = N_{s} + 1, \ldots , 2N_{s} $$
  with $y_{v} \left( i \right)$ the fitting polynomial in segment *v*.
- Determine the *q*th order fluctuation function $F_{q} \left( s \right)$ of the overall segmented intervals *v* of the $2N_{s}$ non-overlapping segments of the Th–K and Th–U distributions:
  5 $$ F_{q} \left( s \right) = \left\{ {\frac{1}{{2N_{s} }}\mathop \sum \limits_{v = 1}^{{2N_{s} }} \left[ {F^{2} \left( {s,v} \right)} \right]^{\frac{q}{2}} } \right\}^{\frac{1}{q}} $$
  where generally, $q \in {{\mathbb{R}}\backslash }\left\{ 0 \right\}$ and *s* ≥ *m* + 2. For *q* = 0, $F_{q} \left( s \right)$ is formulated by:
  6 $$ F_{0} \left( s \right) = exp\left\{ {\frac{1}{{4N_{s} }}\mathop \sum \limits_{v = 1}^{{2N_{s} }} ln\left[ {F^{2} \left( {s,v} \right)} \right]} \right\} $$
  When *q* = 2, the process downgrades to DFA. Generally, the variation of $F_{q} \left( s \right)$ helps to understand the scaling behavior of the data series. Therefore, for different values of *q*, the segregation into $2N_{s}$ intervals, $F^{2} \left( {s, v} \right)$ estimation and $F_{q} \left( s \right)$ determination steps are executed many times for several scale *s* as we want to highlight the scaling behavior of the fluctuation functions Fs of the Th–K and Th–U distributions in the studied OAE3 and non-OAE3 intervals at any scale *s* for different values of *q* .
- Analyze the scaling properties of the fluctuation functions of the Th–K and Th–U distributions through the log–log plots of $F_{q} \left( s \right)$ versus *s* evaluation. If the fluctuation functions $F_{q} \left( s \right)$ and the scaling *s* are positively correlated as a power law defined by Eq. (7), the data series $x_{t}$ of Th–K and Th–U ratios are described as long-range power law correlated.
  7 $$ F_{q} \left( s \right) \sim s^{h\left( q \right)} $$

In Eq. (7), *h*(*q*) defined as the slope of the log(Fs) versus log(*s*) plot represents the generalized Hurst exponent^38^. Analyzing *h*(*q*) helps determine the fractality in the Th–K and Th–U distributions in the studied OAE3 and non-OAE3 intervals. The non-dependence of *h*(*q*) on *q* implies a monofractal nature of the data series while in the case of multifractal dataset, *h*(*q*) is mostly *q* dependence. Furthermore, the *h*(*q*) characterizes the scaling features of the intervals with large variations for positive *q* order (*q* > 0). On the other hand (for negative *q* order, *q* < 0), *h*(*q*) describes the scaling features of the intervals with minor variations. Besides, the generalized Hurst exponent and the mass exponent *τ*(*q*) define a first-order equation expressed by:

8 $$ \tau \left( q \right) = qh\left( q \right) - 1 $$

To characterize the multifractality in the studied OAE3 and non-OAE3 intervals, the singularity spectrum or multifractal spectrum *D*(*α*) of the Th–K and Th–U distributions can be determined via the first-order Legendre transformation

9 $$ \alpha = \left( {d\tau } \right)/\left( {dq} \right)\;{\text{and}} \quad D\left( q \right) = q\alpha - \tau \left( q \right) $$

where $\alpha$ is the multifractal singularity exponent. By using Eq. (9), a relationship exists between the multifractal spectrum *D*(*α*) and the multifractal singularity exponent *α*:

10 $$ \alpha = h\left( q \right) + qh^{\prime}\left( q \right)\;{\text{and}} \quad D\left( \alpha \right) = q\left[ {\alpha - h\left( q \right)} \right] + 1 $$

The degree of multifractality or complexity (also known as the width of the multifractal spectrum) of the data series is related to the dependence of *h*(*q*) on *q*^42^ and is expressed by Eq. (11). Therefore, the degree of multifractality of the Th–K and Th–U distributions in the studied OAE3 and non-OAE3 intervals was explored based on Eq. (11).

11 $$ \Delta \alpha = \alpha_{max} - \alpha_{min} $$

The width of the spectra helps to quantify the strength of the multifractality in the Th–K and Th–U distributions. A narrow spectra width implies weak multifractality in the Th–K and Th–U distributions and vice versa.

## Supplementary information

Supplementary information 1

Supplementary information 2

Supplementary information 3

## Data Availability

The data used in the present article are available in the Supplementary Information files.
